# Hepatoprotective Efficacy and Interventional Mechanism of Qijia Rougan Decoction in Liver Fibrosis

**DOI:** 10.3389/fphar.2022.911250

**Published:** 2022-07-01

**Authors:** Xiao-Feng Chen, Yumei Wang, Shaoxiu Ji, Xin Sun, Quansheng Feng, Han Yu, Chao Liu

**Affiliations:** School of Basic Medical Sciences, Chengdu University of Traditional Chinese Medicine, Chengdu, China

**Keywords:** qijia rougan decoction, liver fibrosis, transcriptomics, transforming growth factor beta, inflammation

## Abstract

Liver fibrosis is a leading contributor to chronic liver diseases such as cirrhosis and liver cancer, which pose a serious health threat worldwide, and there are no effective drugs to treat it. Qijia Rougan decoction was modified from *Sanjiasan*, a traditional Chinese medicine (TCM) described in the “Wenyilun” manuscript. Qijia Rougan decoction possesses hepatoprotective and antifibrotic effects for clinical applications. However, its underlying mechanisms remain largely unknown. In this study, fibrotic rats induced by carbon tetrachloride (CCl_4_) were treated with two doses of Qijia Rougan decoction. Histopathological and serum biochemical analyses were carried out to assess liver structure and function, respectively. High-performance liquid chromatography (HPLC) coupled with mass spectrometry (MS) was performed to identify bioactive compositions in Qijia Rougan decoction. Transcriptome analysis using mRNA-sequencing (mRNA-Seq) was used to explore the underlying mechanisms and verified by quantitative real-time polymerase chain reaction (qRT-PCR) and Western blotting. Qijia Rougan decoction significantly attenuated CCl_4_-induced hepatic fibrotic injury, supported by promoted liver function and improved liver fibrosis. Eight main representative components originating from raw materials in the Qijia Rougan decoction were found to possess an antifibrotic role. Mechanistically, Qijia Rougan decoction regulated biological processes such as oxidation–reduction, fatty acid metabolism, cell adhesion, and transforming growth factor beta (TGFβ) signaling. We determined that Qijia Rougan decoction reversed the expression of inflammatory cytokines and inhibited the activation of fibrosis-related TGFβ signaling. It also reversed the deterioration of liver structure and function in rats induced by CCl_4_. Overall, Qijia Rougan decoction significantly mediated metabolism-associated processes, inhibited inflammatory reactions, and repressed fibrosis-related TGFβ signaling, which prevented liver fibrosis deterioration. Our study deepens our understanding of TCM in the diagnosis and treatment of liver fibrosis.

## Introduction

Liver fibrosis is a collective pathological process present in most chronic liver diseases, including viral hepatitis, alcoholic liver disease (ALD), and non-alcoholic fatty liver disease (NAFLD) (Pellicoro et al., 2014; Schwabe et al., 2020; Friedman and Pinzani, 2022), and is a global health burden causing high morbidity and mortality. Short-term liver fibrosis is considered a reversible and protective reaction to hepatic damage (Kisseleva and Brenner, 2021). Long-term liver fibrosis can lead to cirrhosis and even liver cancer. Liver fibrosis is characterized by excessive deposition of extracellular matrix (ECM) in the liver (Schuppan et al., 2018; Parola and Pinzani, 2019; Xu et al., 2019). The activated hepatic stellate cells (HSCs) primarily account for producing the ECM (Mu et al., 2018). Various injurious stimuli lead to activation of quiescent hepatic stellate cells (HSCs) to yield excessive ECM, which damages the structure and function of the liver (Gao et al., 2020; Chen et al., 2021). Active and effective interventions are expected to delay or prevent the progression of liver fibrosis, prolong patient survival, improve the quality of life, and prevent the deterioration of chronic liver diseases. Thus, reversal or inhibition of liver fibrosis has become a key target in the treatment of liver diseases.

Although there is currently no effective Western medicine for treating liver fibrosis, traditional Chinese medicine (TCM) shows efficient advantages in treating liver fibrosis (Feng et al., 2009; Li, 2020). For example, Xiaoyaosan decoction, containing eight Chinese herbs, was reported to prevent the progression of liver fibrosis induced by CCl_4_ in rats, which was mediated through transforming growth factor beta1 (TGFβ1)/Smad signaling cascades (Zhou et al., 2021). Ganshuang granules, a prepared TCM decoction, can regulate gut microbiota composition and diversity to maintain intestinal integrity and exert antioxidant and anti-inflammatory effects, therefore repressing liver fibrosis (Zhao et al., 2021). Yiguanjian decoction, a representative TCM comprising six medicinal herbs, inhibits liver fibrosis by maintaining the resting state of hepatic stellate cells and hepatocyte viability (Li et al., 2019), repressing liver angiogenesis (Zhou et al., 2015), and modulating inflammatory cytokine release from macrophages (Xu et al., 2021). Due to the multicomponent, multitarget, and multifunction nature of TCM, along with its infrequency of side-effects and drug-resistance induction, the prospect of using TCM to treat liver fibrosis is promising.

Qijia Rougan decoction (QJ) was modified from *Sanjiasan*, found in the “Wenyilun” manuscript and documented to treat lumps in the body during the Ming dynasty. Our previous study demonstrated that another formula, called Fuzheng Xiaozheng prescription (FZXZP), derived from *Sanjiasan*, efficiently inhibited hepatocellular carcinoma (Li X. et al., 2022). Based on the abovementioned research, Qijia Rougan decoction was developed, containing 10 herbs, namely, *Astragalus mongholicus* Bunge, *Angelica sinensis* (Oliv.) Diels, *Trionyx sinensis Wiegmann*, *Eupolyphaga seu* Steleophaga, *Salvia miltiorrhiza* Bunge, *Carthamus tinctorius* L., *Prunus persica* (L.) Batsch, *Sparganium stolonierum*, Buch., *Curcuma phaeocaulis* Valeton, and *Glycyrrhiza uralensis* Fisch. Among these, *Astragalus mongholicus* Bunge was the sovereign drug to enhance qi and has been demonstrated to exert an antifibrotic role (Yang et al., 2008); *Carapax Trionycis*, *Eupolyphaga seu Steleophaga*, *Salvia miltiorrhiza* Bunge, *Carthamus tinctorius* L., and *Prunus persica* (L.) Batsch were ministerial drugs to invigorate blood and nourish Yin and could retard liver fibrosis (Tan et al., 2006; Hu et al., 2017; Li X. et al., 2022); and *Angelica sinensis* (Oliv.) Diels, *Sparganium stolonierum*, Buch., and *Curcuma phaeocaulis* Valeton were adjuvant medicines to expel blood stasis and possessed a hepatoprotective role (Ali et al., 2018). Glycyrrhiza uralensis Fisch. was a conductant drug to regulate medicinal nature. Together, the mixture of Qijia Rougan decoction could reinforce qi, activate blood circulation, remove blood stasis, and dredge collaterals, which together were effective for treating liver fibrosis. Moreover, modern pharmacological studies have shown that almost all the aforementioned traditional Chinese medicines show antifibrotic effects by modulating inflammation and immune responses, inhibiting the activation of hepatic stellate cells (HSCs), and repressing pro-fibrotic signaling pathways (Hu et al., 2017; Ali et al., 2018; Liu and Lv, 2020). However, the role of Qijia Rougan decoction on liver fibrosis and its underlying mechanisms remain to be explored.

In the present study, the rat liver fibrosis model was established by CCl_4_ injection and validated by serum biochemical analyses, liver histopathologic changes, liver collagen, and fibrosis-associated gene expression. We found that Qijia Rougan decoction significantly inhibited CCl_4_-induced liver fibrosis and maintained the integrity of the liver by regulating the expression of inflammation-related genes and modulating transforming growth factor beta (TGFβ) signaling. Collectively, these findings indicate that Qijia Rougan decoction is a promising therapeutic option for treating fibrosis-related liver damage.

## Materials and Methods

### Animals

We purchased 56 healthy male Sprague–Dawley (SD) rats weighing 160–200 g from Chengdu Dossy Experimental Animals Co., Ltd. (Chengdu, China; certification no. SCXK-CHUAN, 2020-030). The rats were kept in a standard 12 h light–dark cycle and allowed free access to water and food. All animal protocols were approved by Ethics Committee of Chengdu University of Traditional Chinese Medicine, and the rat experiments were consistent with the guidelines of the National Institutes of Health (United States).

### Qijia Rougan Decoction Preparation

All herbs were obtained from Hospital of Chengdu University of Traditional Chinese Medicine. Qijia Rougan decoction (QJ) consisted of 10 herbal medicines, including eight herbs and two animal drugs. Information on the herbs is presented in Table 1. We prepared the 10 herbal medicines following the National Pharmacopeia standards. All eight herbs were soaked in purified water for 30 min. The two animal drugs were soaked in purified water for 50 min, and then about 1.5 L of purified water was added and the animal drugs were boiled for 30 min. Then, the eight remaining herbs were added and were allowed to boil for 30 min. The drug solution was filtered, and the abovementioned process was repeated. Finally, the filtered drug solution was concentrated and stored for subsequent use.

**TABLE 1:** The composition of Qijia Rougan decoction.

Chinese name	English name	Botanical name^a^	Family	Weight (g)	Part used	Herbal-producing region
Huang Qi	Astragalus	*Astragalus mongholicus* Bunge	Leguminosae	30	Root	Gansu
Dang Gui	Chiretta	*Angelica sinensis* (Oliv.) Diels	Apiaceae	9	Root	Gansu
Bie Jia	Carapax trionycis	*Trionyx sinensis* Wiegmann	Trionychidae	15	Carapace	Hubei
Tu Bie Chong	Ground beetle	*Eupolyphaga seu* Steleophaga	Polyphagidae	12	Insect body	Anhui
Dan Shen	Salvia miltiorrhiza	*Salvia miltiorrhiza* Bunge	Lamiaceae	18	Root	Shandong
Hong Hua	Safflower	*Carthamus tinctorius* L.	Compositae	18	Flower	Xinjiang
Tao Ren	Semen persicae	*Prunus persica* (L.) Batsch	Rosaceae	18	Seed	Gansu
San Leng	Rhizoma sparganii	*Sparganium stolonierum,* Buch.	Typhaceae	15	Tuber	Anhui
E Zhu	Acruginous turmeric rhizome	*Curcuma phaeocaulis* Valeton	Zingiberaceae	15	Rhizome	Sichuan
Gan Cao	liquorice	*Glycyrrhiza uralensis* Fisch.	Leguminosae	6	Root and rhizome	Xinjiang

a The plant name has been checked with http://www.theplantlist.org.

### Animal Experiments

The rats were randomized to four groups: control group (Control); CCl_4_ group (Model), receiving CCl_4_ from week 0–15; QJ low-dose group (QJ-L), receiving oral QJ (7.0 g/kg) from weeks 9–15 and CCl_4_ from week 0–15; and QJ high-dose group (QJ-H), receiving oral QJ (28.0 g/kg) from weeks 9–15 and CCl_4_ from week 0–15. The rats received CCl_4_ *via* subcutaneous injection of 50% CCl_4_ olive oil twice a week. Then, beginning at week 9, 400 μl QJ was orally administered daily to the QJ-L and QJ-H groups. Finally, the rats were anesthetized, and their serum was obtained through abdominal aortic blood collection. The rats were then euthanized, and their livers were immediately collected for dissection and subsequent analysis.

### Quantitative Real-Time Polymerase Chain Reaction

Total RNA in the rat liver was extracted using TRIzol (Invitrogen). Then, a First-Strand cDNA Synthesis kit (New England Biolabs, #M0277) was used to synthesize the first-strand cDNA. qRT-PCR was carried out with a SYBR Green Master Mix (TaKaRa, #R820A) to detect the relative mRNA levels of the indicated genes. The expressions of *Cxcl12*, *Cxcl1*, *Tgfb1*, *Tgfb2*, *Tgfb3*, *Tgfb1il*, *Col1a1*, and *Timp1* were detected. *Gapdh* was applied as an internal control. Sequences of qRT-PCR primers are shown in Table 2. Gene expression was calculated by the 2^−ΔΔCT^ method.

**TABLE 2:** mRNA primer sequences.

Gene names	Forward primer (5′-3′)	Reverse primer (5′-3′)
*Cxcl12*	TGT​TGA​CCC​CGG​ACA​CCT​AA	TGGCGCCCAAGGGAAT
*Cxcl1*	CAG​ACA​GTG​GCA​GGG​ATT​CA	CCTGGCGGCATCACCTT
*Tgfb1*	GAAACGGAAGCGCATCGA	CTG​GCG​AGC​CTT​AGT​TTG​GA
*Tgfb2*	TCA​TCC​CAA​ATA​AGA​GCC​AAG​AG	TGG​AGG​TGC​CAT​CGA​TAC​CT
*Tgfb3*	GCG​CCC​CCT​CTA​CAT​TGA​C	GGT​TCG​TGG​ACC​CAT​TTC​C
*Tgfb1i1*	CGCTTCTCCCCACGATGT	AAG​GCG​GTG​ACC​ATT​TTA​TGT​C
*Col1a1*	GTA​CAT​CAG​CCC​AAA​CCC​CA	CAG​GAT​CGG​AAC​CTT​CGC​TT
*Timp1*	GAC​ACG​CTA​GAG​CAG​ATA​CCA	CCA​GGT​CCG​AGT​TGC​AGA​AA
*Gapdh*	GTG​ATG​GGT​GTG​AAC​CAC​GAG	GTC​ATG​AGC​CCT​TCC​ACG​ATG

### Histological Staining

Fresh rat livers were fixed in 4% paraformaldehyde. Fixed livers were embedded in paraffin and then cut into 5-µm sections. Liver sections were stained with hematoxylin–eosin (H&E) or Masson’s staining to identify liver structure and the state of collagen accumulation, respectively. A quantitative digital image analysis system (Image-Pro Plus 6.0) was used to analyze the histological staining images.

### Immunohistochemical Staining

For immunohistochemical (IHC) staining, paraffin-embedded liver sections were deparaffinized with xylene and rehydrated with a gradient of ethanol. Endogenous peroxidases were quenched by 3% hydrogen peroxide (H_2_O_2_) for half an hour. EDTA-Tris (pH 8.0) was used to boil the sections for 3 min for antigen retrieval. The sections were blocked with goat serum to block nonspecific staining of the liver and incubated with individual primary antibodies α-SMA (Servicebio, GB111364) and collagen I (Servicebio, GB11022) overnight at 4°C. Finally, diaminobenzidine (DAB) staining was performed and counterstained with hematoxylin.

### Western Blotting

The protein was isolated with RIPA lysis buffer (Beyotime, #P0013B) containing a protease inhibitor cocktail and phosphatase inhibitor as previously described (Tang et al., 2017). The liver homogenate was sonicated and centrifuged at 4°C for 15 min to collect the supernatant for Western blotting. Then, the concentration of every supernatant protein was evaluated by the BCA Protein Assay Reagent (Thermo, #23227). Eventually, the supernatant protein was mixed with SDS-PAGE Sample Loading Buffer (Beyotime Biotechnology, #P0015) and boiled in water to denature the protein. 20 μg of protein was subjected to SDS-PAGE and transferred to a PVDF membrane. Five percent non-fat milk was used to block the PVDF membrane. Then, the membrane was incubated with individual primary antibodies overnight at 4°C. Subsequently, the membrane was incubated with a horseradish peroxidase–conjugated secondary antibody (ZSGB-BIO; #ZB2301, #ZB2305), and protein expression was detected by Pierce ECL Western Blotting Substrate (Thermo, #32106). Antibodies to p-SMAD3 (CST, 9520), SMAD3 (Proteintech, 66516-1-Ig), p-SMAD2 (CST, 3108), SMAD2 (CST, 5339), TGF-βR1 (Servicebio, GB11271), TGF-βR2 (Servicebio, GB112244), TGF-β (bioworlde, BS1361), and GAPDH (abcam, ab8245) were used in this study.

### HPLC-MS/MS Analysis

High-performance liquid chromatography (HPLC) tandem mass spectrometry (MS) was performed for quality control. Briefly, the Qijia Rougan decoction was mixed with 1 ml of methanol: water (4:1, v/v), vortexed for 10 min, and centrifuged for 10 min at 4°C and 20,000 *g*. The supernatant was filtered through a 0.22-μm filter membrane for subsequent HPLC analysis (UltiMate 3000 RS; Thermo Scientific). The analytical column was AQ-C18 with a dimension of 150 mm × 2.1 mm and 1.8 μm particle size (Welch). The mobile phase contained the aqueous phases of 0.1% formic acid and an organic phase of methanol. The chromatography gradients are shown below: 2% methanol at 1 min, 20% methanol at 5 min, 50% methanol at 10 min, 80% methanol at 15 min, 95% methanol at 20 min, 95% methanol at 25 min, 2% methanol at 26 min, and 2% methanol at 30 min. The column was maintained at 35°C. The injection volume was 5 μl, and the flow rate was 0.30 ml/min. MS analysis was carried out on a Q Exactive high-resolution mass spectrometer (Thermo Fisher) with an ESI Source. The MS conditions were spray voltage: 3.8 kV (positive); capillary temperature: 300°C; aux gas heater temperature: 350°C; scanning mode: positive and negative ion switching scanning; scan range: 150.0–2000.0 m/z; resolution: 70,000 full mass, 17,500 dd-MS2; detection mode: full mass/dd-MS2.

### Serum Biochemistry Analysis

The concentrations of serum alanine aminotransferase (ALT), aspartate aminotransferase (AST), and total bilirubin (TBIL) were detected with a fully automated biochemical analyzer (Chemray 800, Rayto) according to the manufacturer’s instructions in Servicebio. Alanine aminotransferase (ALT, cat. no. GM1102), aspartate aminotransferase (AST, cat. no. GM1103), and total bilirubin (TBIL, cat. no. GM1105) were purchased from Servicebio. Enzyme-linked immunosorbent assay (ELISA) was used to detect the content of hyaluronic acid (HA), laminin (LN), and type III procollagen (PC-III) in the blood, according to the manufacturer’s instructions.

### mRNA Sequencing

Total RNA was extracted from liver tissues using TRIzol reagent, as previously described (Tang et al., 2017). A Bioanalyzer 2,100 system (Agilent Technologies, CA, United States) was used to assess RNA integrity. Novogene performed mRNA sequencing. Total RNAs were used as the input material for the RNA sample preparations to prepare the transcriptome sequencing libraries. The clustering of the index-coded samples was performed on a cBot Cluster Generation System, using a TruSeq PE Cluster Kit v3-cBot-HS (Illumina), according to the manufacturer’s instructions. After cluster generation, the library preparations were sequenced on an Illumina NovaSeq platform, and 150-bp paired-end reads were generated. Raw data (raw reads) in a fastq format were first processed through in-house Perl scripts. In this step, clean data (clean reads) were obtained by removing reads containing adapters, reads containing ploy-N, and low-quality reads from the raw data. In addition, Q20, Q30, and GC contents were calculated. All downstream analyses were based on clean high-quality data. A corrected *p*-value <0.05 and an absolute fold change ≥1.5 were set as the thresholds for detecting significantly different expression levels. Raw sequence data in this study can be found on the National Center for Biotechnology Information (NCBI) database (https://www.ncbi.nlm.nih.gov/) and are available through accession number (PRJNA826993).

### Statistical Analysis

All data were presented as mean ± SEM. One-way analysis of variance (ANOVA) was performed to analyze significant differences between the control, model, QJ-L, and QJ-H. *p* values less than 0.05 were considered statistically significant. All statistical analyses were carried out using GraphPad Prism 8.0 or SPSS Version 21 software.

## Results

### Chemical Components of Qijia Rougan Decoction

**FIGURE 1:**
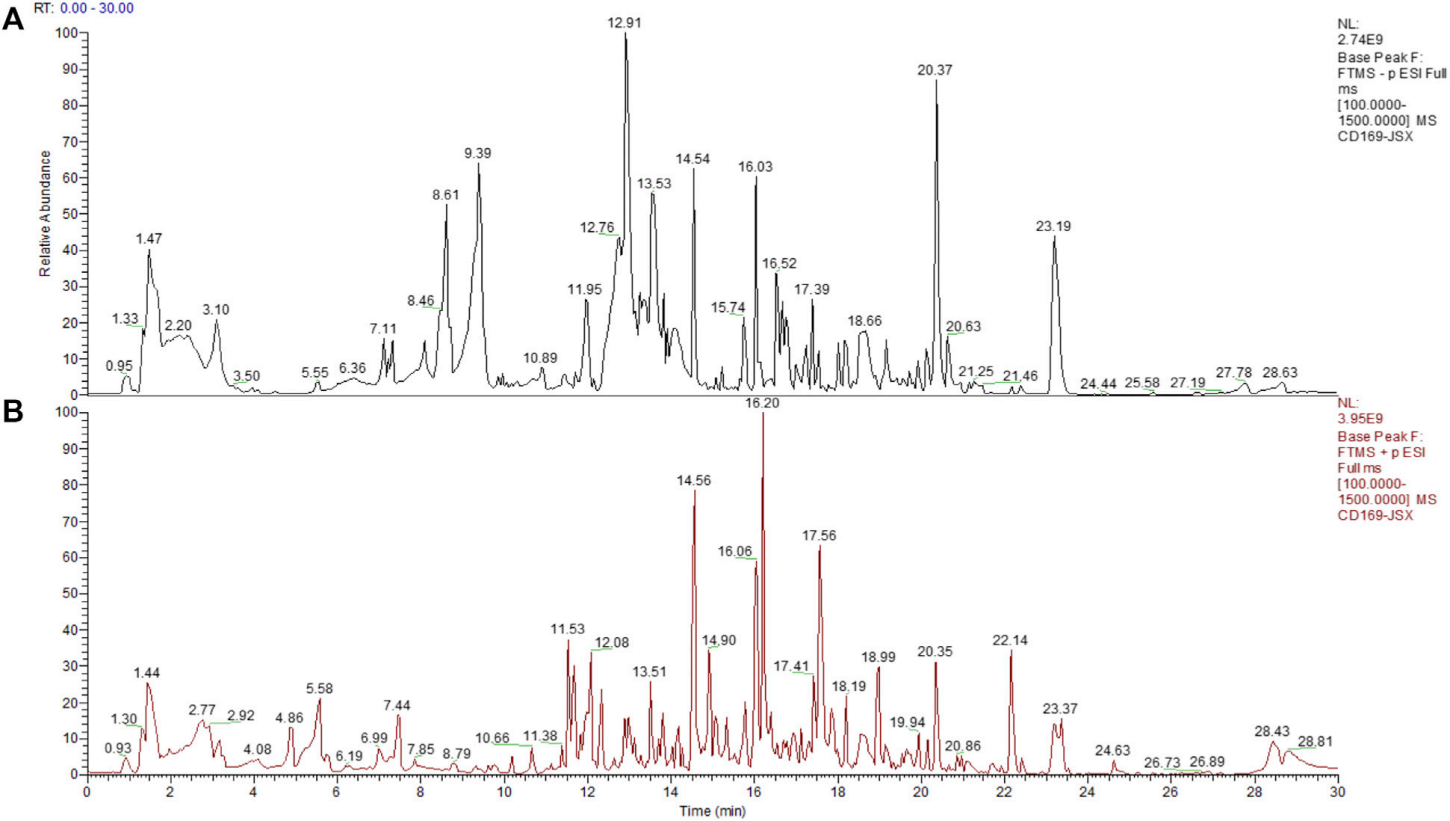
Total ion current chromatograms (TICCs) of the Qijia Rougan decoction (QJ). **(A)** TICCs of the negative (ESI−) ionization modes of the QJ; **(B)** TICCs of the positive (ESI+) ionization modes of the QJ.

In order to preliminary identify the constituents of Qijia Rougan decoction and explore its antifibrotic role, HPLC-MS/MS analysis was implemented to identify the bioactive components of Qijia Rougan decoction in this study. The total ion current chromatograms (TICCs) of the negative and positive ionization modes of Qijia Rougan decoction are shown in Figures 1A,B, respectively. Overall, we identified 1028 components, with 425 components with a >60 mzCloud best-match score in the mzCloud database. We focused on the eight main compounds originating from raw materials in Qijia Rougan decoction with high mzCloud best-match scores >95, namely, ursolic acid, tanshinone IIA, isoliquiritigenin, formononetin, 18-β-glycyrrhetinic acid, cryptotanshinone, daidzein, and nicotinic acid (Table 3). Interestingly, the eight main compounds had antifibrotic roles. For example, ursolic acid from *Salvia miltiorrhiza* Bunge and *Glycyrrhiza uralensis* Fisch was reported to inhibit HSC activation, inflammasome pathways, and bacterial dysbiosis, which eventually reversed liver fibrosis progression (Yu et al., 2017; Nie et al., 2021). Formononetin from *Astragalus mongholicus* Bunge, *Glycyrrhiza uralensis* Fisch, and *Sparganium stolonierum* Buch was demonstrated to be an efficient component against liver fibrosis (Wang et al., 2021). Compound characteristics are presented in Table 3, mainly including mzCloud Best-Match score, formula, molecular weight, retention time, area, and classification. Overall, these identified compounds accounted for the potential antifibrotic effects of Qijia Rougan decoction.

**TABLE 3:** Main compounds in Qijia Rougan decoction.

Name	mzCloud best match	Formula	Molecular weight	RT [min]	Area (max.)	Classification
Ursolic acid	97.6	C_30_H_48_O_3_	456.36035	22.161	2876158.66968326	Pentacyclic triterpenoid
Tanshinone IIA	97.5	C_19_H_18_O_3_	294.12493	19.92	67272425.0540303	Diterpenoid naphthoquinone
Isoliquiritigenin	97.4	C_15_H_12_O_4_	256.07273	11.975	112889882.437397	Flavonoid
Formononetin	97.2	C_16_H_12_O_4_	268.07302	16.041	269361987.926751	Isoflavone
18-β-Glycyrrhetinic acid	96.9	C_30_H_46_O_4_	470.33886	18.693	19747998.2703174	Triterpene saponin
Cryptotanshinone	96.8	C_19_H_20_O_3_	296.14049	18.947	181443226.025542	Diterpene
Daidzein	96.3	C_15_H_10_O_4_	254.05765	14.25	10265683.3609214	Isoflavones
Nicotinic acid	95.6	C_6_H_5_NO_2_	123.03215	2.293	47518158.8212902	Organic acid

RT, retention time.

### Qijia Rougan Decoction Improves the Liver Index, Liver Function, and Serum Liver Fibrosis Index of CCl_4_-Induced Rats

**FIGURE 2:**
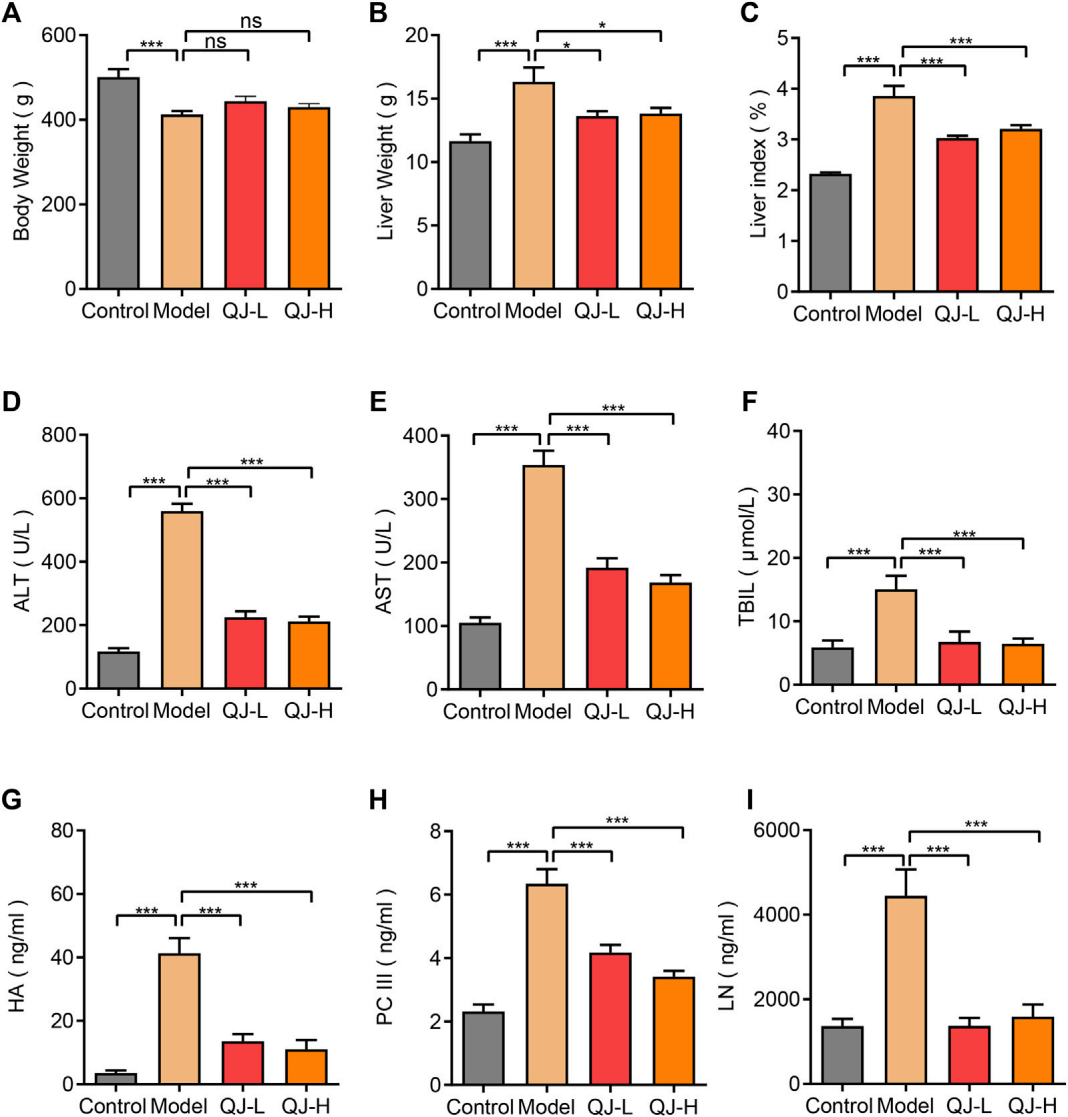
Detection of liver index and serum biochemical indicators of rats in four groups. **(A)** Body weight (*n* = 8–16); **(B)** Liver weight (*n* = 8–16); **(C)** Liver index = Liver weight/Body weight X 100% (*n* = 8–16); **(D)** ALT: alanine aminotransferase (*n* = 8–16); **(E)** AST: aspartate aminotransferase (*n* = 8–16); **(F)** TBIL: total bilirubin (*n* = 8–16); **(G)** HA: hyaluronic acid (*n* = 8–10); **(H)** PC III: type III procollagen (*n* = 8–10); **(I)** LN: laminin (*n* = 8–10). *p < 0.05 and ***p < 0.001. ns, not significant.

To identify the effects of Qijia Rougan decoction on liver index and serum biochemical indicators of fibrotic hepatic damage, we treated CCl_4_-induced hepatic fibrosis rats with different Qijia Rougan decoction dosages (7.0 and 28 g/kg). The results showed that CCl_4_ decreased the body weight and increased the liver weight of rats compared with that of controls (Figures 2A,B). Although Qijia Rougan decoction did not improve the body weight of rats induced by CCl_4_, it decreased their liver weight (Figures 2A,B). Similarly, Qijia Rougan decoction improved the liver index of CCl_4_-treated rats (Figure 2C).

To validate the effect of Qijia Rougan decoction on CCl_4_-induced rat liver function, we detected the abundance of ALT, AST, and TBIL in the serum of rats. We observed a strong increase in ALT, AST, and TBIL levels in the blood of CCl_4_-induced rats, which was dramatically reduced by oral Qijia Rougan decoction treatment (Figures 2D–F). These findings indicated that Qijia Rougan decoction repressed the CCl_4_-induced increase of serum liver function indicators.

To further investigate the antifibrotic role of Qijia Rougan decoction, we detected HA, PC-III, and LN levels in the serum. Consistent with the serum liver function index, HA, PC-III, and LN serum levels were markedly increased in response to CCl_4_. However, these indexes were prominently reduced upon Qijia Rougan decoction treatment (Figures 2G–I). These results demonstrated that Qijia Rougan decoction alleviated the CCl_4_-induced increase in serum liver fibrosis indicators. Collectively, these findings indicated that Qijia Rougan decoction improved the CCl_4_-induced liver indexes and serum biochemical indicators.

### Qijia Rougan Decoction Protects the Liver From Injury Induced by CCl_4_ in Rats

**FIGURE 3:**
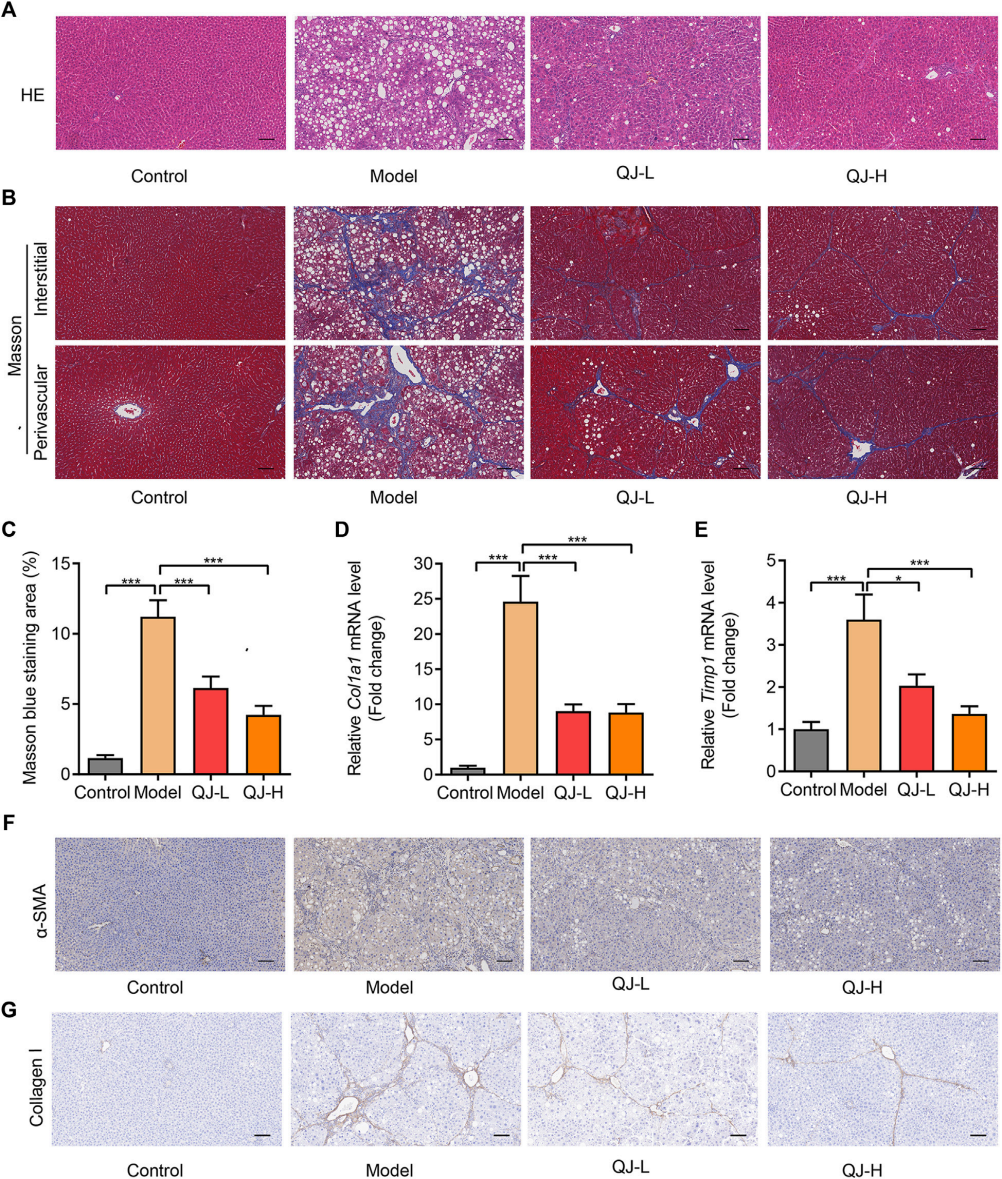
Qijia Rougan decoction protects the rat liver from CCl_4_-induced injury. **(A)** Hematoxylin and eosin (H&E, scale bar = 100 µm) staining was performed to detect liver structure in rats; **(B)** Masson’s staining (scale bar = 100 µm) was performed to detect liver fibrosis in rats; **(C)** Quantification of liver fibrosis in four groups (*n* = 8–10); **(D,E)** qRT-PCR was used to analyze *Col1a1* and *Timp1* mRNA levels in rats (*n* = 6–12); **(F,G)** Immunohistochemical (IHC) staining was performed to detect the level of α-SMA and collagen I in the rat liver. *p < 0.05 and ***p < 0.001.

To further evaluate the antifibrotic function of Qijia Rougan decoction, we measured the liver histopathological changes. Hematoxylin–eosin (H&E) staining was performed to detect the liver structure. Our results indicated significantly swollen and necrotic hepatocytes and inflammatory infiltration in CCl_4_-induced rats when compared with controls. Conversely, these abnormities were reversed in the liver of Qijia Rougan decoction–treated rats when compared with the models (Figure 3A). In addition, Masson’s staining showed that CCl_4_ notably increased the formation of perivascular and interstitial fibrosis, while Qijia Rougan decoction alleviated these phenotypes (Figures 3B,C). Moreover, the mRNA expression of hepatic fibrosis markers, such as *Col1a1* and *Timp1*, was significantly increased in CCl_4_-induced rats and repressed by Qijia Rougan decoction treatment (Figures 3D,E). Furthermore, HSC activation is a hallmark of liver fibrosis, and α-SMA is a symbol of activated HSCs (Tsuchida and Friedman, 2017). Immunohistochemical (IHC) staining demonstrated that α-SMA levels were markedly enhanced upon CCl_4_ treatment compared to those in the control group, while Qijia Rougan decoction treatment significantly decreased them (Figure 3F), indicating that Qijia Rougan decoction can efficiently repress HSC activation. In addition, liver fibrosis is characterized by excessive deposition of extracellular matrix in the liver, and collagen I is the major extracellular matrix component. IHC staining results indicated that CCl_4_ induction promoted the protein expression of collagen I, which was dramatically reduced by Qijia Rougan decoction treatment (Figure 3G), indicating that Qijia Rougan decoction significantly suppressed extracellular matrix formation. Collectively, these findings suggested that Qijia Rougan decoction inhibited liver fibrosis progression.

### Gene Expression Analysis by mRNA-Seq

**FIGURE 4:**
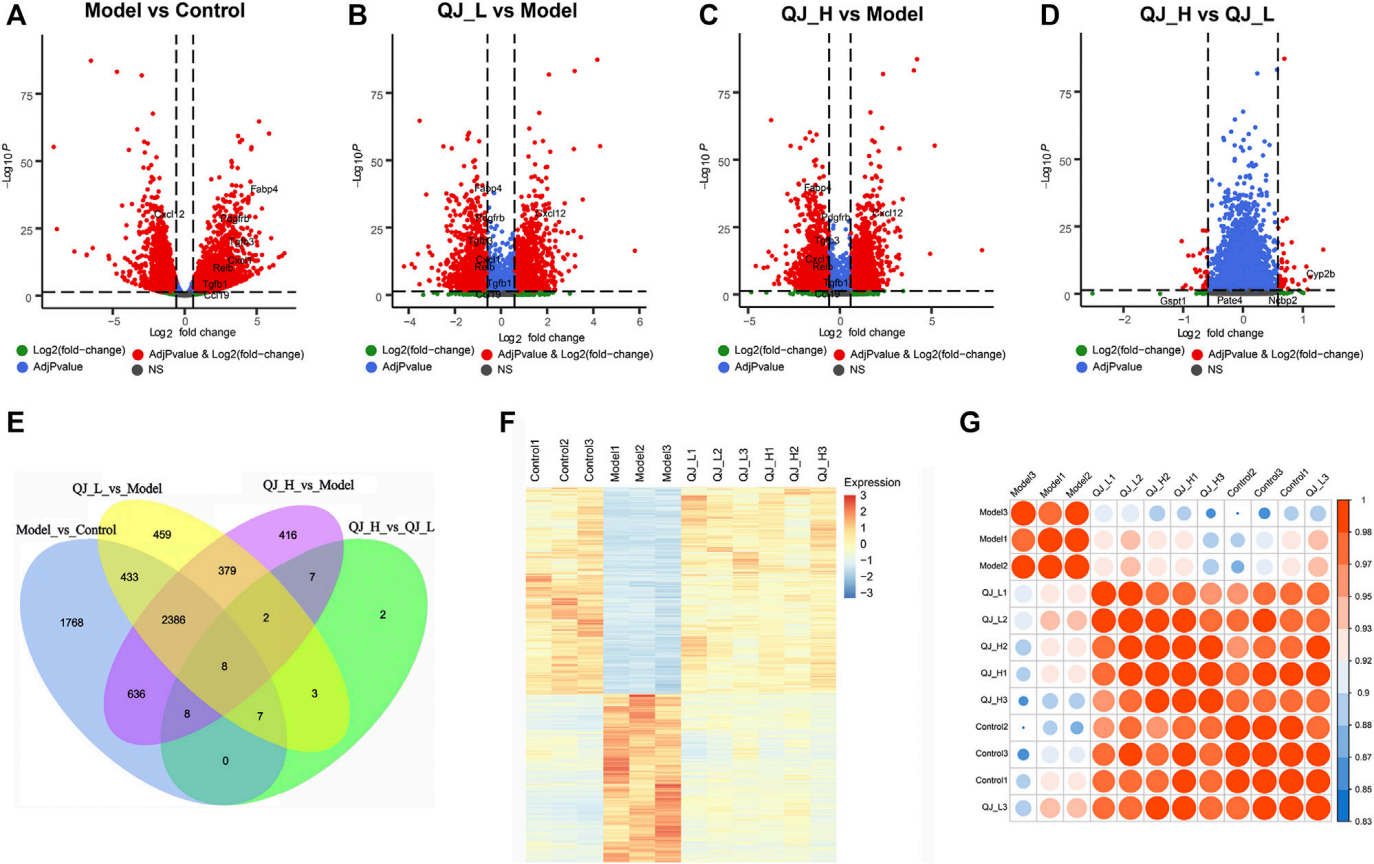
DEG analysis of the mRNA-Seq. **(A–D)** Volcano plot of differentially expressed genes in Qijia Rougan decoction–treated rats with fibrosis. **(A)** Model vs. Control; **(B)** QJ-L vs. Model; **(C)** QJ-H vs. Model; **(D)** QJ-H vs. QJ-L; **(E)** Numbers of the overlapping genes in different comparisons (Venn); **(F)** Expression heatmap in the four experimental groups; **(G)** Corrplot correlation heat map analyses between the four groups.

To gain insight into liver transcriptional changes in response to Qijia Rougan decoction treatment, we performed mRNA-seq. We obtained 17294 genes, and differentially expressed genes (DEGs) were filtered based on *p*-value <0.05 and absolute foldchange ≥1.5. We detected 2,346 upregulated genes and 2,900 downregulated genes between the model and control groups, 1,969 upregulated genes and 1,708 downregulated genes in the comparison of QJ-L versus the model group, and 1,963 and 1,879 upregulated and downregulated genes, respectively, between QJ-H and the model group (Figures 4A–C; Table 4). Surprisingly, there were only 37 DEGs in comparison between the QJ-H and QJ-L groups, including 24 upregulated genes and 13 downregulated genes (Figure 4D; Table 4). The overlapping genes in the different groups are shown in Figure 4E. Interestingly, the expression tendencies of DEGs could be reversed by Qijia Rougan decoction treatment (Figure 4F). Notably, corrplot correlation heat map analyses indicated that Qijia Rougan decoction treatment groups were closely correlated with the control group (Figure 4G). These data suggested the potential therapeutic benefits of Qijia Rougan decoction.

**TABLE 4:** Differentially expressed genes in the different comparison groups.

Comparison group	Upregulated	Percentage (%)	Downregulated	Percentage (%)	Total DEGs
Model vs. control	2346	44.7	2900	55.3	5246
QJ-L vs. model	1969	53.5	1708	46.5	3677
QJ-H vs. model	1963	51.1	1879	48.9	3842
QJ-H vs. QJ-L	24	64.9	13	35.1	37

QJ-L, Qijia Rougan decoction low-dose group; QJ-H, Qijia Rougan decoction high-dose group; DEGs, differential expressed genes.

### Target Gene Analysis of Qijia Rougan Decoction

**FIGURE 5:**
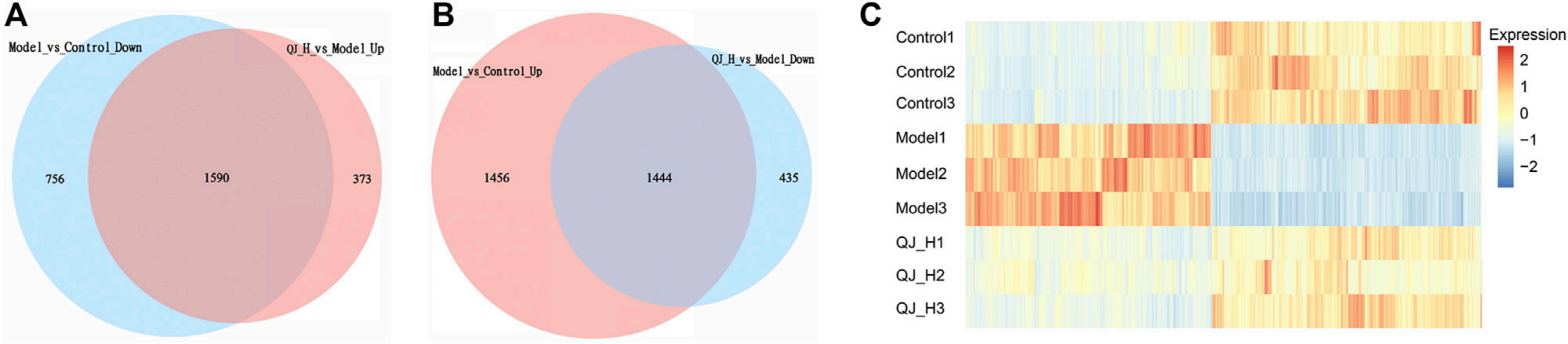
DEG Target analysis of QJ-H. **(A)** Overlapping DEGs between Model vs. Control-down and QJ-H vs. Model-up (Venn); **(B)** Overlapping DEGs between Model vs. Control-up and QJ-H vs. Model-down (Venn); **(C)** Heatmap for hierarchical cluster analysis of DEGs between Control, Model, and QJ-H.

To further elucidate the underlying action model of Qijia Rougan decoction (QJ) in liver fibrosis, we focused on the DEG target of QJ-H. Upregulated DEG target of QJ-H referred to the overlapping genes between Model-vs-Control-Down and QJ-H-vs-Model-Up, which were downregulated in the model group whereas upregulated in the QJ-H group, and the downregulated DEG target of QJ-H referred to the overlapping genes between Model-vs-Control-Up and QJ-H-vs-Model-Down, which were highly expressed in the model group whereas lowly expressed in the QJ-H group. We identified 1,590 upregulated and 1,444 downregulated DEG targets in QJ-H (Figures 5A,B). Surprisingly, the abovementioned DEG target showed remarkable differences between groups, and the disturbance induced by CCl_4_ in the model group could be rebalanced by Qijia Rougan decoction in the QJ-H group (Figure 5C). Similarly, 1,482 upregulated and 1,341 downregulated DEG targets in QJ-L were identified (Supplementary Figures S1A,B), and the expression tendencies of DEG targets in QJ-L were consistent with those in QJ-H (Supplementary Figure S1C).

### Gene Ontology and Kyoto Encyclopedia of Genes and Genomes Enrichment Analysis on Differentially Expressed Gene Target of Qijia Rougan Decoction

**FIGURE 6:**
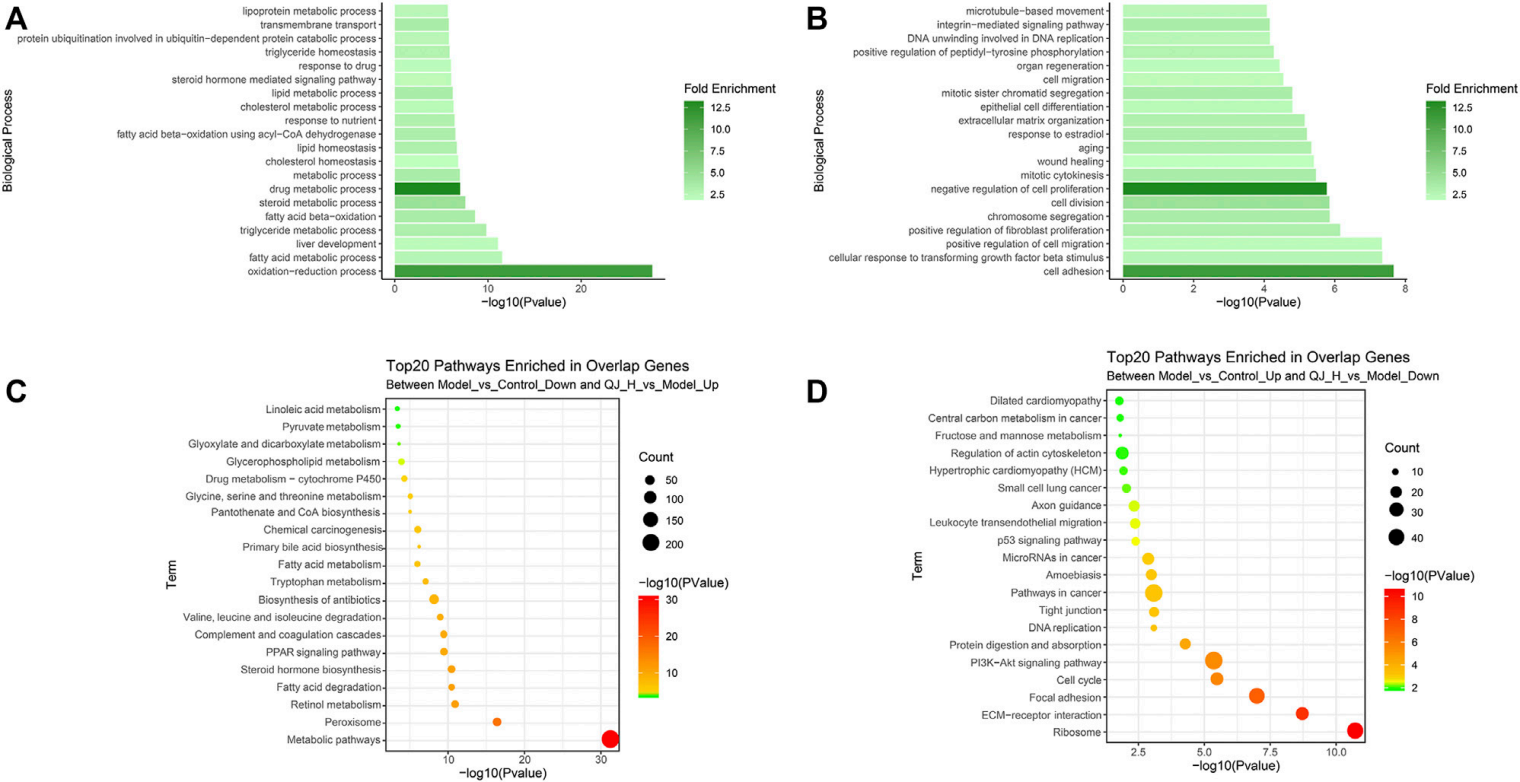
GO and KEGG enrichment analysis of the DEG target of QJ-H. **(A)** GO analysis histogram of the 1590 overlapping upregulated DEGs between Model vs. Control-down and QJ-H vs. Model-up; **(B)** GO analysis histogram of the 1444 overlapping downregulated DEGs between Model vs. Control-up and QJ-H vs. Model-down; **(C)** KEGG enrichment analysis of the 1590 overlapping upregulated DEGs between Model vs. Control-down and QJ-H vs. Model-up; **(D)** KEGG enrichment analysis of the 1444 overlapping downregulated DEGs between Model vs. Control-up and QJ-H vs. Model-down.

To clarify the functions and signaling cascades enriched upon Qijia Rougan decoction treatment in CCl_4_-induced liver fibrosis, 1,590 upregulated and 1,444 downregulated DEG targets of QJ-H were used for Gene Ontology (GO) and Kyoto Encyclopedia of Genes and Genomes (KEGG) enrichment analysis. GO analysis indicated that upregulated DEG targets of QJ-H were involved in the oxidation–reduction process, fatty acid metabolism, liver development, and triglyceride metabolism (Figure 6A). Downregulated DEG targets of QJ-H primarily participated in biological processes including cell adhesion, TGFβ signaling, positive regulation of cell migration, and positive regulation of fibroblast proliferation (Figure 6B). KEGG analysis suggested that upregulated DEG targets of QJ-H were closely related to metabolism pathways including peroxisome, retinol metabolism, fatty acid degradation, and steroid hormone biosynthesis (Figure 6C). Conversely, downregulated DEG targets of QJ-H were mainly associated with ribosome, ECM–receptor interactions, focal adhesions, and the cell cycle (Figure 6D). GO and KEGG enrichment analysis results of DEG target in QJ-L were similar to those in QJ-H (Supplementary Figures S2A–D).

### Qijia Rougan Decoction Molecular Mechanism Analysis

**FIGURE 7:**
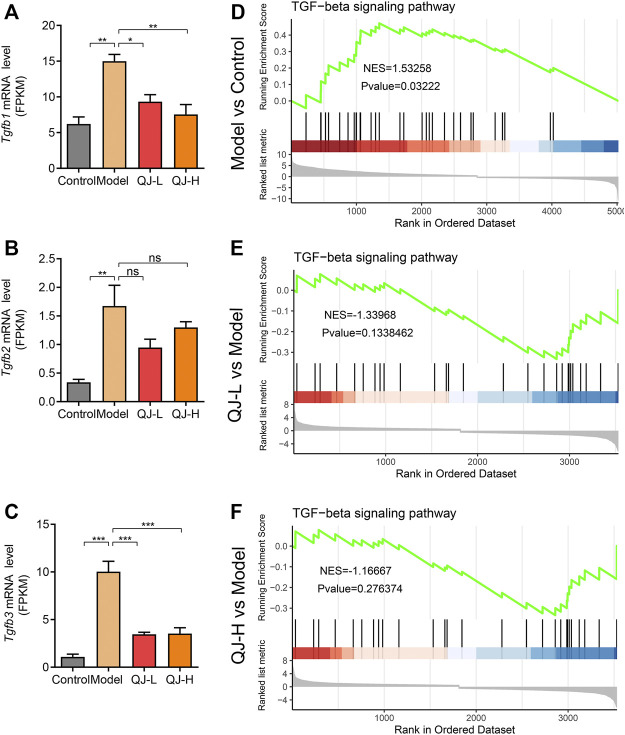
Expression level and Gene Set Enrichment Analysis (GSEA) analysis of TGF-beta signaling. **(A–C)** Bulk RNA-seq analysis of the expression level of *Tgfb1*, *Tgfb2*, and *Tgfb3* (*n* = 3); **(D–F)** Gene Set Enrichment Analysis (GSEA) analysis of TGF-beta signaling. NES, normalized enrichment score; positive and negative NESs indicate higher and lower expression, respectively. *p < 0.05, **p < 0.01, and ***p < 0.001. ns, not significant.

Accumulating evidence has demonstrated the profibrogenic effect of TGFβ signaling (Fabregat et al., 2016; Sun et al., 2021). In GO enrichment analyses, we observed the significant enrichment of TGFβ in the downregulated DEG target of QJ (Figure 6B; Supplementary Figure S2B). Bulk RNA-seq analyses also showed the increased expression of three TGFβ isoforms (*Tgfb1*, *Tgfb2*, and *Tgfb3*), especially the *Tgfb1* and *Tgfb3*, after CCl_4_ injection, along with its decrease upon Qijia Rougan decoction treatment (Figures 7A–C). To further explore the role of TGFβ signaling, we also analyzed the differential expression of TGFβ signaling pathway components between CCl_4_ and Qijia Rougan decoction treatment using Gene Set Enrichment Analysis (GSEA) based on the abovementioned data. Notably, we found that TGFβ signaling was remarkably activated in response to CCl_4_ (Figure 7D), whereas low- or high-dose Qijia Rougan decoction inhibited it (Figures 7E,F). These data indicated that Qijia Rougan decoction exerted a protective role in liver fibrosis, at least in part, through TGFβ signaling.

**FIGURE 8:**
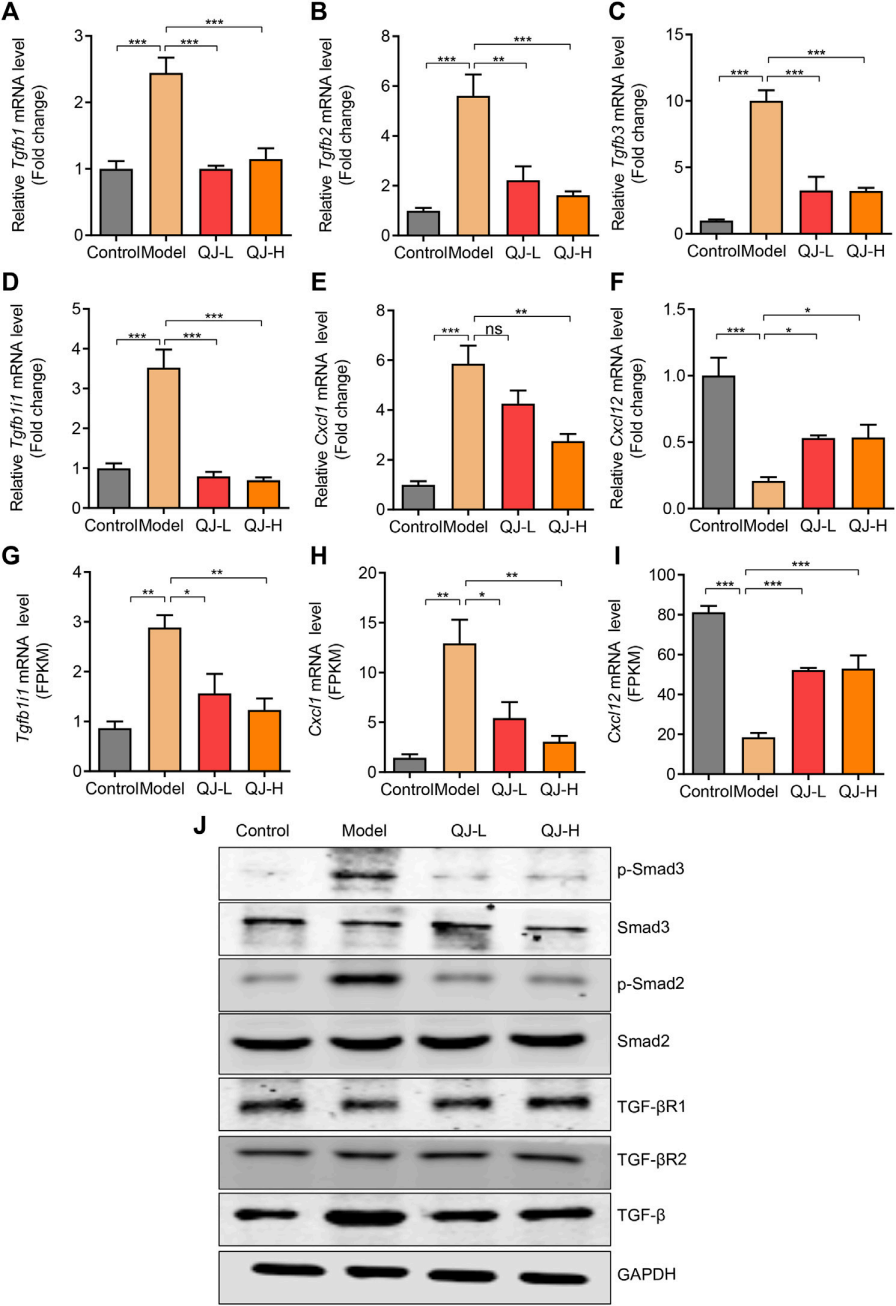
Validation of the expression of mRNA and proteins. **(A–F)** qRT-PCR validations of related genes (*n* = 4); **(A)** *Tgfb1*: transforming growth factor beta 1; **(B)** *Tgfb2*: transforming growth factor, beta 2; **(C)** *Tgfb3*: transforming growth factor beta 3; **(D)** *Tgfb1i1*: transforming growth factor beta 1–induced transcript 1; **(E)** *Cxcl1*: C-X-C motif chemokine ligand 1; **(F)** *Cxcl12*: C-X-C motif chemokine ligand 12; **(G–I)** Bulk RNA-seq analysis of the expression level of *Tgfb1i1*, *Cxcl1*, and *Cxcl12* (*n* = 3); **(J)** Representative Western blotting was performed to detect the levels of p-Smad3, Smad3, p-Smad2, Smad2, TGF-βR1, TGF-βR2, and TGF-β.

To validate our results, we detected the expression of several genes associated with the TGFβ signaling pathway using qRT-PCR. Consistent with mRNA-seq results, three TGFβ isoforms (*Tgfb1*, *Tgfb2*, and *Tgfb3*) showed increased expression in the model group, and Qijia Rougan decoction could decrease their levels (Figures 8A–C). The expression pattern of *Tgfb1i1*, a gene induced by TGFβ, was similar to that of TGFβ isoforms (Figures 8D,G). Furthermore, Western blotting results showed Qijia Rougan decoction did not influence the expression of TGF-β receptors I and II. But CCl_4_ injection induced the activation of the TGF-β and its classical downstream Smad2 and Smad3 proteins, while Qijia Rougan decoction could inhibit the activation of the TGFβ signaling pathway (Figure 8J). In addition, the inflammatory response is an important contributor to liver fibrosis. Therefore, we also detected inflammatory factor expression and found that Qijia Rougan decoction could also modulate inflammatory responses, supported by increased inflammatory *Cxcl1* cytokine levels and decreased inflammatory *Cxcl12* chemokine levels in CCl_4_-induced rats, which were reversed by Qijia Rougan decoction (Figures 8E,F,H,I). Collectively, these findings indicated that Qijia Rougan decoction protected the fibrotic liver by inhibiting TGFβ signaling and modulating inflammation responses.

## Discussion

Owing to a lack of effective drugs to treat liver fibrosis, diseases associated with liver fibrosis have become a serious health threat worldwide. TCM holds great potential to tackle this health burden. In the present study, we found that Qijia Rougan decoction exerted a hepatoprotective role in liver fibrosis. We found that CCl_4_ significantly enhanced the formation of liver fibrosis, manifesting as large numbers of apoptotic hepatocytes, collagen accumulation, severe inflammatory infiltration, and the activation of inflammatory factors and inflammatory signaling pathways. Oral Qijia Rougan decoction attenuated these phenotypes by inhibiting TGFβ signaling and inflammatory responses. These data suggest the therapeutic effect of Qijia Rougan decoction on liver fibrosis.

Patients subjected to early liver fibrosis generally have no symptoms, while its early detection and treatment could strongly prevent its deterioration to cirrhosis and hepatic carcinoma, improving the survival and quality of life of patients (Zhang and Friedman, 2012). Clinicians could diagnose a patient with liver fibrosis based on liver biopsy in the preceding decades (Liu et al., 2019). Due to its invasiveness and sample error, the non-invasive detection of hepatic fibrosis is more practical (Wong, 2018; Liu et al., 2019). ALT, AST, and TBIL are important indicators for assessing liver function (Qiao and Zhao, 2021). Qijia Rougan decoction was shown to inhibit the increased levels of serum ALT, AST, and TBIL induced by CCl_4_, indicating that it significantly protected liver integrity. In addition, the degree of liver fibrosis is also classified by the abundance of serum liver fibrosis indicators, including HA, LN, PCIII, and IV-C (Ma et al., 2021; Qiao and Zhao, 2021). Qijia Rougan decoction was demonstrated to reverse the enhanced HA, LN, and PCIII levels, suggesting its antifibrotic effects. We conclude that Qijia Rougan decoction is a hepatoprotective TCM formula.

TCM preparations often show multiple components and multiple targets (Wu et al., 2021; Xue et al., 2021). Over 1,000 ingredients were identified in the Qijia Rougan decoction *via* HPLC-MS/MS analysis. Many ingredients derived from raw herbs and animal drugs in the Qijia Rougan decoction were reported to possess antifibrotic effects. For example, formononetin, a common component of *Astragalus mongholicus* Bunge, *Glycyrrhiza uralensis* Fisch., and *Sparganium stolonierum,* Buch., was proven to be efficient against liver fibrosis (Wang et al., 2021). It also inhibited cholestasis, a precursor of liver fibrosis, partly by increasing the expression of SIRT1 to activate the PPARα, which eventually maintained bile acid metabolism and reduced inflammatory reactions (Yang et al., 2019). These data support the antifibrotic effect of Qijia Rougan decoction. Furthermore, ursolic acid, a pentacyclic triterpenoid, rich in *Salvia miltiorrhiza* Bunge and *Glycyrrhiza uralensis* Fisch*.*, found in the Qijia Rougan decoction, reportedly possessed excellent antioxidative, anti-inflammatory, and antifibrotic effects (Wan et al., 2019; Nie et al., 2021). A series of published studies have demonstrated the significant protective role of ursolic acid against liver fibrosis and mechanical insights ranging from the inhibition of hepatic stellate cell (HSC) activation (Wang et al., 2011; Yu et al., 2017), hepatic circadian rhythm regulation (Kwon et al., 2018), and gut dysbiosis improvement (Wan et al., 2019; Wan et al., 2020) to NADPH oxidase 4 (NOX4)/NOD-like receptor protein 3 (NLRP3) inflammasome pathway suppression (Nie et al., 2021) and oxidative stress repression through LKB1-AMPK signaling activation (Yang et al., 2015). Another main component derived from *Salvia miltiorrhiza* Bunge, called Tanshinone IIA, was also reported to have therapeutic potential for treating liver fibrosis (Ying et al., 2019), as it might inhibit HSC proliferation and extracellular matrix deposition (Shi et al., 2020). More interestingly, other main components identified in Qijia Rougan decoction, such as isoliquiritigenin, glycyrrhetinic acid, and daidzein, were demonstrated to have excellent antifibrotic activity (Chen et al., 2013; Soumyakrishnan et al., 2014; Wang et al., 2021). Therefore, the components present in Qijia Rougan decoction may synergistically mediate its hepatoprotective functions.

TGFβ, a central contributor of fibrogenesis, has been widely studied in liver fibrosis (Fabregat et al., 2016; Sun et al., 2021). TGF-β1, TGF-β2, and TGF-β3 belong to the TGFβ superfamily, and all play essential roles in fibrotic disease pathogenesis (Sun et al., 2021). In general, TGF-β1 is the most widely and deeply studied subtype in the formation of liver fibrosis (Fan et al., 2019; Xu et al., 2020). It is considered that activation of TGF-β1 could accelerate the progression of liver fibrosis by activating hepatic stellate cells (HSCs) (Li Y. et al., 2022). Activated HSCs are characterized by increased α-SMA expression. Mechanistically, TGFβ activates HSCs and increases extracellular matrix (ECM) deposition, which promotes liver fibrosis (Dewidar et al., 2019). In our study, we observed increased levels of α-SMA induced by CCl_4_ which decreased upon Qijia Rougan decoction treatment. Meanwhile, collagen I, the dominating component of the extracellular matrix, was prominently improved by Qijia Rougan decoction. The canonical TGFβ signaling pathway is dependent on the downstream of Smad proteins, including Smad2 and Smad3 (Dewidar et al., 2019; Chen et al., 2021). Surprisingly, in this study, the canonical TGFβ signaling pathway was activated along with an enhancement of three TGFβ isoforms (*Tgfb1*, *Tgfb2*, and *Tgfb3*) in the fibrotic liver. Upon Qijia Rougan decoction treatment, TGFβ signaling was repressed and the expression of *Tgfb1*, *Tgfb2*, and *Tgfb3* decreased. Collectively, TGFβ signaling inhibition may be a core mechanism underlying the Qijia Rougan decoction–mediated hepatoprotective functions.

In conclusion, we demonstrated the value of Qijia Rougan decoction as a hepatoprotective TCM. Qijia Rougan decoction prevented CCl_4_-induced hepatocyte damage and fibrotic liver injury by modulating inflammatory responses and TGFβ signaling. These results indicate that Qijia Rougan decoction and its bioactive components are potential therapeutic options for treating liver fibrosis in clinical practice.

## Data Availability

The datasets presented in this study can be found in online repositories. The names of the repository/repositories and accession number(s) can be found below: https://www.ncbi.nlm.nih.gov; PRJNA826993.
