# Supplementary material for: Hepatoprotective Efficacy and Interventional Mechanism of Qijia Rougan Decoction in Liver Fibrosis
Source: Front Pharmacol. 2022 Jul 1;13:911250. doi: 10.3389/fphar.2022.911250 (PMC9283647; doi:10.3389/fphar.2022.911250)
Supplement: Supplementary file 1 [file DataSheet1.DOCX]

Supplementary Material


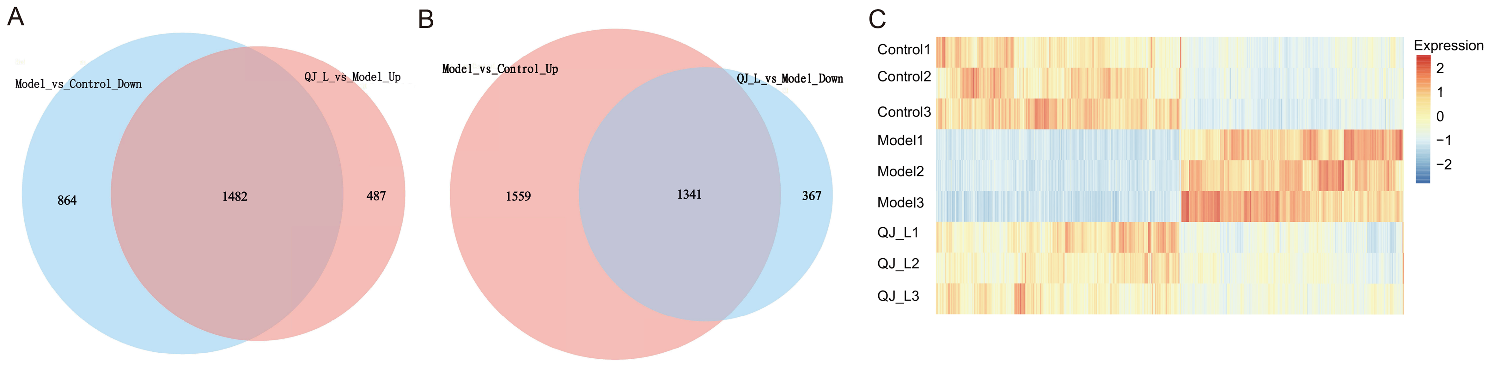


**Figure. S1.** **Target DEGs analysis of QJ-L.** (A) The overlapped of DEGs between Model vs Control-down and QJ-L vs Model-up (Venn); (B) The overlapped of DEGs between Model vs Control-up and QJ-L vs Model-down (Venn); (C) Heat map for hierarchical cluster analysis of DEGs between Control, Model, and QJ-L.


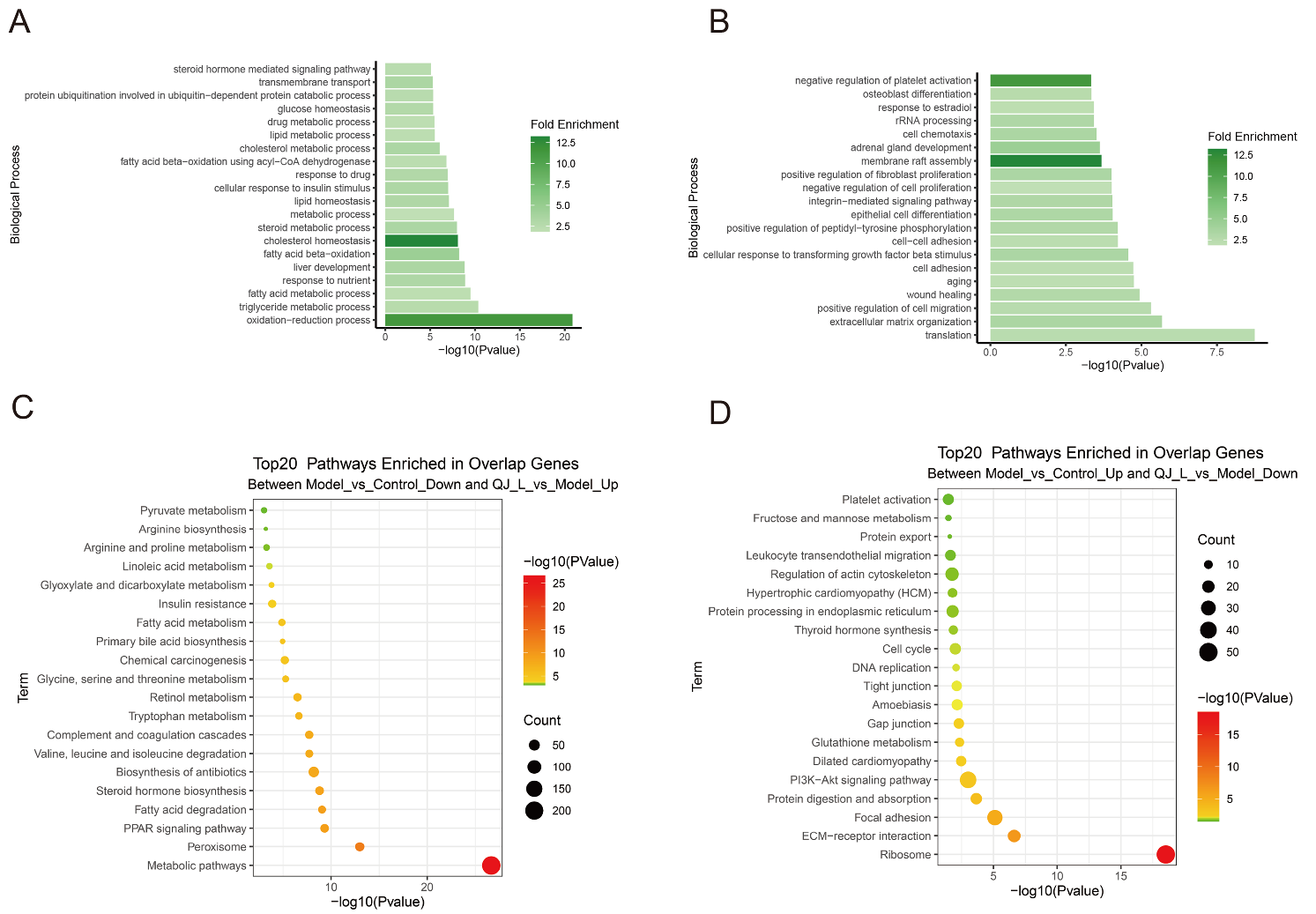


**Figure. S2. GO and KEGG enrichment analysis of target DEGs of QJ-L.** (A) GO analysis histogram of the 1482 overlapped up-regulated DEGs between Model vs Control-down and QJ-L vs Model-up; (B) GO analysis histogram of the 1341 overlapped down-regulated DEGs between Model vs Control-up and QJ-L vs Model-down; (C) KEGG enrichment analysis of the 1482 overlapped up-regulated DEGs between Model vs Control-down and QJ-L vs Model-up; (D) KEGG enrichment analysis of the 1341 overlapped down-regulated DEGs between Model vs Control-up and QJ-L vs Model-down.
